# Metabolites Alterations in the Medial Prefrontal Cortex of Methamphetamine Users in Abstinence: A ^1^H MRS Study

**DOI:** 10.3389/fpsyt.2018.00478

**Published:** 2018-10-15

**Authors:** Qiuxia Wu, Chang Qi, Jiang Long, Yanhui Liao, Xuyi Wang, An Xie, Jianbin Liu, Wei Hao, Yiyuan Tang, Baozhu Yang, Tieqiao Liu, Jinsong Tang

**Affiliations:** ^1^Department of Psychiatry, The Second Xiangya Hospital, Central South University, Changsha, China; ^2^Mental Health Institute, The Second Xiangya Hospital, Central South University, Changsha, China; ^3^Chinese National Clinical Research Center on Mental Disorders, Second Xiangya Hospital, Central South University, Changsha, China; ^4^National Clinical Research Center on Mental Disorders, Changsha, China; ^5^National Technology Institute on Mental Disorders, Changsha, China; ^6^Hunan Key Laboratory of Psychiatry and Mental Health, Changsha, China; ^7^Department of Psychological Sciences, Texas Tech University, Lubbock, TX, United States; ^8^Department of Radiology, Hunan Provincial People's Hospital, Changsha, China; ^9^Department of Psychiatry, Yale University School of Medicine, New Haven, CT, United States

**Keywords:** methamphetamine, mPF, NAA/PCr+Cr, Glu/PCr+Cr, mI/PCr+Cr, ^1^H MRS

## Abstract

**Background:** The medial prefrontal cortex (mPFC) contains various neurotransmitter systems and plays an important role in drug use. Broad body of literature on how methamphetamine (MA) affects the structure and metabolism in the animal's mPFC is emerging, while the effects on metabolites of mPFC among human is still unclear. In this study, proton magnetic resonance spectroscopy (1H MRS) was used to measure metabolites of mPFC in methamphetamine dependent subjects.

**Methods:** Sixty-one subjects with a history of MA dependence (fulfiled the Diagnostic and Statistical Manual of Mental Disorders, fourth edition criteria) and 65 drug-naïve control subjects (age19–45) completed 1H MRS scans using 3.0T Siemens MRI scanner. Single voxel spectra were acquired from the mPFC bilaterally using a point resolved spectroscopy sequence (PRESS). The 1H MRS data were automatically fit with linear combination model for quantification of metabolite levels of n-acetyl-aspartate (NAA), myo-inositol (mI), glycerophosphocholine plus phosphocholine(GPC+PC), phosphocreatine plus creatine (PCr+Cr), and glutamate (Glu). Metabolite levels were reported as ratios to PCr+Cr.

**Results:** The MA group showed a significant reduction in NAA/PCr+Cr ratio and elevation in Glu/PCr+Cr ratio and mI/PCr+Cr ratio, compared with healthy control. No significant correlation was found between metabolite ratios and MA use variables.

**Conclusions:** MA use is associated with a significant increased Glu/PCr+Cr ratio, mI/PCr+Cr ratio and reduced NAA/PCr+Cr ratio in the mPFC of MA dependence subjects. These findings suggest that Glu may play a key role in MA induced neurotoxicity.

## Introduction

Methamphetamine is one of the most consumed amphetamine-type stimulants (ATS) worldwide. According to the World Drug Report 2017 (1), global methamphetamine seizures reached a new peak of 132 tons, increased 21% than previous year, accounting for 60–80% of ATS seizures annually. As MA use is spreading and the treatment demand is growing, evidence on effective treatment is scarce while MA represents the greatest global health burden among ATS (1). There is abundant evidence that MA cause long-lasting impairment to the brain both in preclinical and clinical research. In preclinical studies, chronic MA exposure activated microglials (2) and astrocytes, and increased inflammatory mediators and other oxidative stress related factors (3). While in human neuroimaging studies demonstrated that chronic MA use leads to serious brain changes, including dopaminergic (4, 5), monoaminergic (6), and serotoninergic (7, 8) neurotransmitter system, cerebral glucose metabolism (9, 10), structure and integrity (9–11).

^1^H magnetic resonance spectroscopy (^1^H MRS) provides an invasive method to explore the metabolites in the brain. Previous ^1^H MRS research on MA dependent (MAD) subjects shows alterations in n-acetyl-aspartate (NAA) (12–18), choline (Cho) (12, 13, 16, 17, 19), myo-inositol (mI) (13, 14), and glutamate (Glu) or Glx (meaning glutamate+glutamine) (15, 20) concentrations or the ratios of these metabolites to creatine (Cr). Most of these studies focused on anterior cingulate (12, 16–18), basal ganglia (13, 19), frontal gray (13) and white matter (13–15), with less evidence in the medial prefrontal cortex (mPFC).

The mPFC is a terminal region of the mesocorticolimbic dopamine system which has been reported to modulate reward seeking behavior (21, 22) and is associated with drug addiction (23). The functional connectivity of the mPFC is decreased in various mental disorders (24–26), including addiction (27, 28). Previous studies suggested ATS dependent subjects have smaller volume (29–31) and decreased gray matter density (32) in the mPFC. To date, there is no report on measuring metabolite levels in the mPFC in MA users relative to healthy subjects. The mPFC contains pyramidal glutamatergic neurons that project to numerous regions (33) and repeated amphetamine administration alters a-amino-3-hydroxy-5-methylisoxazole-4-propionic acid (AMPA) receptor subunits mRNA levels in rat mPFC (34). Thus, metabolite levels in the mPFC may be different between methamphetamine dependent (MAD) subjects and healthy chontrols, especially Glu. In this present study, we performed a semi-quantitative analysis to quantify the levels of NAA, mI, GPC+PC, and Glu of the mPFC in MAD subjects. In this study, we aimed to investigate whether MA use significantly altered metabolite ratios to PCr+Cr in the mPFC. It was hypothesized that first, MA use would be associated with altered metabolite levels in the mPFC, and second, the altered metabolite levels would be significantly correlated with MA use variables, including age of first MA use (years old), duration of MA use (months), and duration of abstinence from MA use (days). In addition, we explored possible relationship between cigarettes smoked per day (CPD), Beck Depression Inventory (BDI) score, State-Trait Anxiety Inventory (STAI) scores and the metabolite levels in MA users.

## Method

### Subjects

The data were collected as a part of the brain imaging study on methamphetamine-induced psychotic symptoms, a study hosted at the Second Xiangya Hospital of Central South University. One hundred and twenty-six subjects (61 MAD subjects and 65 drug-free healthy subjects, age 19–45) were enrolled in this study. Subjects between 18 and 45 years were entitled to participate in the study. MAD volunteers were recruited from The Kangda Voluntary Drug Rehabilitation Centers in Hunan Province. All MA users fulfiled the Diagnostic and Statistical Manual of Mental Disorders, fourth edition (DSM-IV) criteria (35) for lifetime MA dependence assessed by the Structured Clinical Interview (SCID) (36). MAD subjects were excluded if they met criteria for other substance dependence (excluding nicotine dependence) at any time. Subjects were required to abstain from MA for at least 48 h before scanning. Drug free healthy control subjects were recruited from community through advertising. Participants were excluded if they (i) had any general medical condition or neurological disorder, including infectious, hepatic or endocrine disease; (ii) had a history of severe head injury with skull fracture or loss of consciousness of more than 10 min; (iii) had any current or previous psychiatric disorder; (iv) had a family history of psychiatric disorder; (v) women during pregnant or breast-feed stage; (vi) had contraindications for MRI. Two licensed psychiatrist, at MD level, conducted all clinical interviews. Subjects were fully informed about the measurement and MRI scanning in the study. Written informed consent was given by all subjects. This study was approved by the Ethics Committee of the Second Xiangya Hospital, Central South University (No. S095, 2013) and was carried out in accordance with the Declaration of Helsinki. We used the BDI-II to measure the depression symptoms in the last week before undergoing MRI scans. STAI was used to measure anxiety level before MRI scanning (STAI-Y1), and anxiety level as a personal characteristic (STAI-Y2). The demographic characteristic were shown in Table 1.

**Table 1:** Demographics of participants and MA use variables in MRS study.

	**Abstinent MAD subjects (*N* = 61)**	**Controls (*N* = 65)**	***P***
Age (range)	29.0 ± 5.8 (19–40)	30.0 ± 6.1 (21–45)	0.38
Gender(female/male)	54/7	53/12	0.33
Education(years)	11.5 ± 2.9	12.4 ± 2.6	0.07
BMI	24.0 ± 3.2	22.8 ± 3.2	0.08
CPD	20 (10, 20)	16 (5, 20)	< 0.01
BDI Score	11.0 ± 7.8	4 (0, 8)	< 0.01
Total AI Score	75.6 ± 16.0	71.5 ± 16.7	0.17
STAI-Y1	35.0 ± 8.0	33.5 ± 9.9	0.37
STAI-Y2	40.6 ± 9.9	38.0 ± 8.8	0.12
Age started using MA (years old)	23.9 ± 5.7		
Duration of MA use (months)	51.2 ± 26.8		
Abstinence from using MA(days)	42.7 ± 20.9		

### Magnetic resonance data acquisition

**Figure 1:**
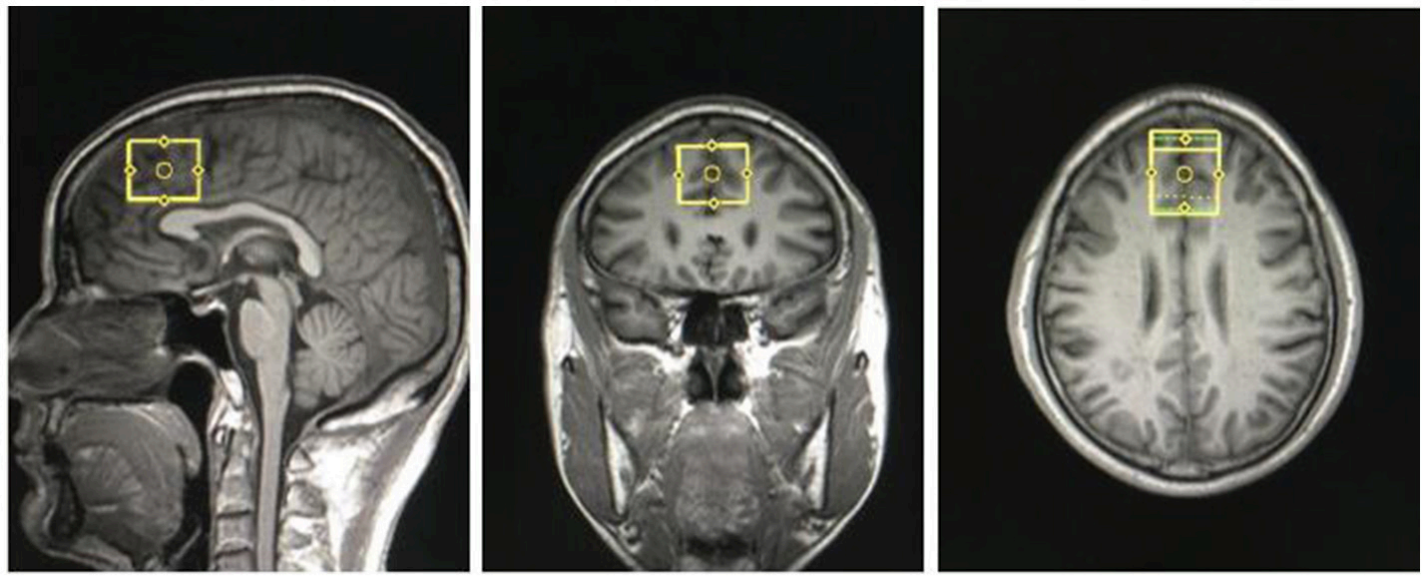
Region of interest in medial prefrontal cortex in coronal, sagittal and transverse views.

Structural MRI and MRS data were acquired with a Siemens Magnetom Trio 3.0 T MR scanner (Siemens, Erlangen, Germany) using an eight-channel standard quadrature headcoil at the Magnetic Resonance Center of Hunan provincial People's Hospital, China. Three-dimensional T1-weighted images were collected using a gradient echo sequence (repetition time = 2,000 ms, echo time = 2.26 ms, field of view = 256 × 256 mm, flip angle = 8°, matrix size = 256 × 256, number of slices = 176, slice thickness = 1 mm). Using these images, a single ^1^H MRS voxel was placed on the corpus callosumand centered on the intrahemispheric fissure, including medial superior frontal gyrus and anterior cingulate cortices, not containing the orbitofrontal cortex (see Figure 1). ^1^H MRS was performed using a short-echo point resolved spectroscopy sequence (PRESS; repetition time = 1,500 ms; echo time = 30 ms; voxel size 30 × 25 × 30 mm; number of scans = 256, spectral bandwidth = 1,200 Hz, the number of data points = 1,024 points). Water suppression was achieved using a chemical shift selective (CHESS) sequence.

**Figure 2:**
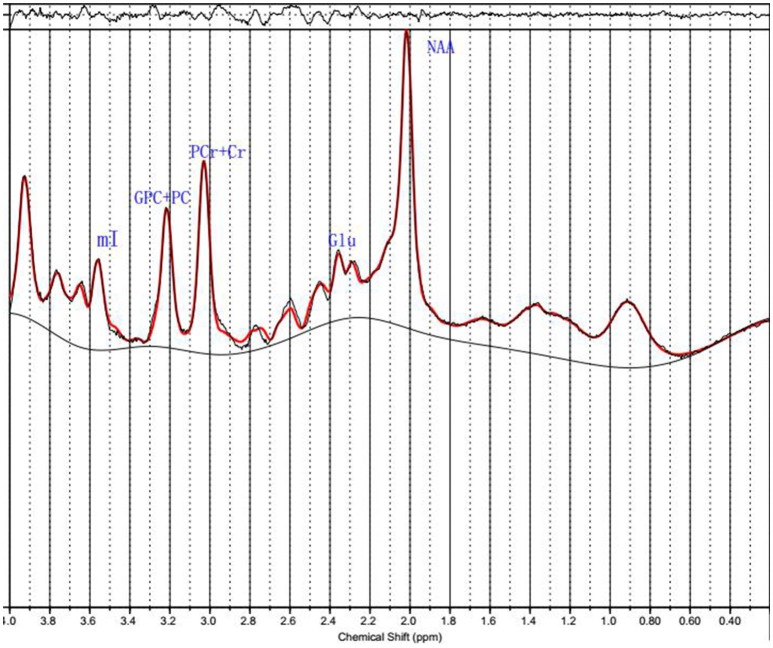
^1^H MRS data.Spectra of the unfiltered data superimposed with the LCModel fit.

^1^H MRS spectra were automatically fit with linear combination model (LCModel version 6.3–1B [LCMODEL Inc. CA (37)] at the Second Affiliated Hospital, Shantou University Medical College located in Shantou, Guangdong, China. Metabolite concentration for NAA, PCr+Cr, GPC+PC, mI and Glu were acquired using LCModel software (Figure 2). The signal-to-noise ratio (S/N) and full width at half maximum (FWHM) of each spectrum were checked for quality to ensure they were adequate for reliable peak fitting for the metabolites of interest. Only those spectra with FWHM ≤ 0.1 ppm and S/N ≥ 20 were retained. Furthermore, only those metabolite speaks satisfying the LCModel criterion less than 20% of Cramer-Rao lower bound (CRLB) value were reported here. PCr+Cr severs as a reference for other metabolite peaks on the assumption that its concentration is relatively constant. We first observed that there was no absolute PCr+Cr difference prior to forming ratios with respect to PCr+Cr. The common practice of normalization by PCr+Cr removd the across-subject variability, which arises from technical factors, such as coil loading. From these data, the metabolite ratios of NAA/PCr+Cr, mI/PCr+Cr, Glu/PCr+Cr, and GPC+PC/PCr+Cr were reported here.

Segmentation was performed on T1-weighted images using New Segment+DARTEL in Data Processing & Analysis of Brain Imaging (38). Estimation of tissue volume was collected from the normalized gray matter, white matter, and cerebro-spinal fluid (CSF) images using custom Matlab (The Mathworks, Inc.) code (http://www.cs.ucl.ac.uk/staff/G.Ridgway/vbm/get_totals.m). GM fractions, WM fractions and CSF fractions in the MAD subjects and controls were shown in Table 2. No statistical significance was found between the two groups.

**Table 2:** Tissue fraction of the region of interest in the mPFC.

	**Abstinent MAD subjects (*N* = 61)**	**Controls (*N* = 65)**	***P***
Gray matter fraction	0.44 ± 0.08	0.46 ± 0.01	0.06
White matter fraction	0.30 ± 0.06	0.32 ± 0.02	0.10
CSF fraction	0.23 ± 0.05	0.23 ± 0.01	0.85

### Statistical analysis

Statistical analysis was performed with SPSS 20 (IBM Inc. New York, USA). Assumption of normality of each variable was tested with the Shapiro–Wilk test. Because of non-normality of the data, CPD and BDI scores were compared using a Mann-Whitney U test, and gender with Chi-square test for independence. Metabolite concentrations were reported as mean ± standard deviation. General Linear Model multivariate analysis was used to evaluate group differences in metabolite ratios controlling for CPD and gray matter tissue fraction in the voxle. Correlation analyses between metabolite ratios and each of clinical parameters, including age, months of MA use, days of abstinence, age of onset of MA use, CPD, BDI, total STAI scores, STAI-Y1 score, and STAI-Y2 score were performed using Pearson's or Spearman's correlation analysis, followed by Bonferroni test. Statistical significance was defined at *p* < 0.05, two-tailed.

## Results

### Demographic characteristic

The groups did not differ in gender, mean age, BMI, or years of education (Table 1). The CPD (*p* < 0.01) in MAD subjects was higher than those in healthy controls. The BDI score (*p* < 0.01) was higher in MAD subjects. There were no significant differences in the total STAI scores (*p* = 0.17), STAI-Y1 (*p* = 0.37), or STAI-Y2 (*p* = 0.12) between the two groups.

### Tissue composition within voxels of interest

Segmentation indicates the fractional contribution of gray matter, white matter and CSF in the MAD group = 44% gray, 30% white and 23% CSF and in healthy control group = 46% gray, 32% white and 23% CSF. No differences in the fractions of gray (*p* = 0.06), white (*p* = 0.10), and CSF (*p* = 0.85) were detected between MAD subjects and controls.

### FWHM, S/N, and CRLB values of MRS data

There was no difference between the MAD group and control group in FWHM (*p* = 0.23). There was significant difference in S/N between MAD group and healthy control group (*p* < 0.01). There were significant differences between the two groups in CRLB values for NAA (*p* < 0.01), mI (*p* < 0.01), GPC+PC (*p* = 0.01) and Glu (*p* < 0.01) (see Table 3).

**Table 3:** FWHM, S/N, and CRLB values between MAD subjects (n = 61) and healthy controls (*n* = 65).

	**Abstinent MAD subjects (*N* = 61)**	**Controls (*N* = 65)**	***P***
FWHM(ppm)	0.05 (0.05, 0.06)	0.06 (0.048, 0.067)	0.23
S/N	28.85 ± 5.01	37.91 ± 7.96	< 0.01
CRLB values for NAA	0.03 (0.03, 0.04)	0.02 (0.02, 0.03)	< 0.01
CRLB values for mI	0.04 (0.03, 0.04)	0.03 (0.03, 0.04)	< 0.01
CRLB values for GPC+PC	0.02 (0.02, 0.03)	0.02 (0.02, 0.02)	0.12
CRLB values for Glu	0.06 (0.05, 0.06)	0.05 (0.05, 0.06)	< 0.01

### ^1^H MRS metabolite ratios

The MAD group and control group did not differ significantly in absolute PCr+Cr values (mean = 6.47 vs. 6.58, *p* = 0.44), which served as the denominator for the ratios tested. Compared with healthy controls, MAD subjects had significant decreased NAA/PCr+Cr ratios (mean = 1.12 vs. 1.17, *p* = 0.02), increased mI/PCr+Cr ratios (mean = 0.85 vs. 0.80, *p* < 0.01) and Glu/PCr+Cr ratios (mean = 1.03 vs. 0.95, *p* < 0.01) in the mPFC. There was not significant difference in GPC+PC/PCr+Cr between the two groups (mean = 0.27 vs. 0.27, *p* = 0.73) (see Table 4).

**Table 4:** Metabolite concentrations in the mPFC of MAD subjects (*n* = 61) and healthy controls(*n* = 65).

	**Abstinent MAD subjects (*N* = 61)**	**Controls (*N* = 65)**	***P***
PCr+Cr	6.47 ± 0.83	6.58 ± 0.70	0.44
NAA/PCr+Cr	1.12 ± 0.08	1.17 ± 0.07	0.01
mI//PCr+Cr	0.85 ± 0.09	0.80 ± 0.09	< 0.01
GPC+PC/PCr+Cr	0.27 ± 0.03	0.27 ± 0.03	0.78
Glu/PCr+Cr	1.03 ± 0.15	0.95 ± 0.14	< 0.01

### ^1^H-MRS metabolite levels with MA use, age, CPD, and anxiety

For the MA users, there were no significant correlation between ratios of metabolites and MA use variables. There were no significant correlation between age, CPD and metabolite ratios.

## Discussion

There was no significant difference in PCr+Cr levels between MAD group and control group, so the differences in NAA/PCr+Cr, mI/PCr+Cr, and Glu/PCr+Cr between groups are probably due to the differences in NAA, mI and Glu levels between groups. The first finding of the present study is that the NAA/PCr+Cr ratio was increased in the mPFC of MAD subjects. Finding the decreased ratio of NAA/PCr+Cr among MAD subjects is consistent with previous studies (12, 16, 18, 39, 40) and as well as other psychiatric disorders (41). NAA is taken as a neuronal marker, and reflects neuronal integrity, viability and number (42). NAA plays a key role in enhancing mitochondrial energy production from Glu (42) and also reflects functional status of neuronal mitochondria (43). The changes of NAA/PCr+Cr may reflect adverse neuron function disorder. Besides, the pathological deletion of dendrite or degeneration of neuron may be related with decreased NAA. The present finding suggests that there is decreased neuronal integrity, viability, number, or mitochondraial dysfunction in the mPFC in MAD individuals, even though they have been abstinent from MA.

The second finding is that the ratio of mI/PCr+Cr was significantly increased in the mPFC of MAD subjects compared with healthy controls. The increased ratio of mI/PCr+Cr in MAD group is in line with previous studies, which were reported in frontal gray matter and white matter of MA users (13, 14, 44). Mostly mI is considered as a marker of glial (45). Some studies suggested that Ins is an osmoregulator (46) and contributes to glucose storage (47) and is a precursor in the PI-cycle second messenger system (47). Elevation of mI may suggest that proliferation of glial cells or inflammation due to the damage of neurons induced by methamphetamine, which is a marker of MA induced neurons damage after NAA decreased. MA has been reported to induced gliosis *in vitro* and in animal experiment. After acute administration of MA to rats (48) and vervet monkeys (49) induced glial activation, and gliosis remains after stopped exposure one and half a year (49).

The final finding is a significant increase in ratios of Glu/PCr+Cr in the mPFC of MAD subjects. The most consistent alteration across MA abuse was reduction in NAA, while change in Glu was inconsistent. Glx and Glu were reported to be lower in the mPFC (39), precuneus, posterior cingulate, and right inferior frontal cortex (20), while Glu was repored to be higher in frontal white matter of abstinent MAD subjects (15). But the Glu levels in the posterior gray matter did not differ with HC (15). In one study, in the frontal gray matter of the MA users, the Glx concentration reduced during early abstinence, reached relatively normal after 1–2 months, and exceeded normal levels in longer-term abstinence (50). In the Crocker's study, the concentration levels of Glu in the MA group was reduced relative to HC and schizophrenia patients (39). In another study, however, Glu levels in the ACC and DLPFC did not differ between MAD subjects and controls (12). Several variables may contribute to these inconsistencies. These studies evaluated different brain regions and used different field strength. Other variables include the length of time using MA, dose, frequency of use, duration of abstinence and the sample size.

This is a relatively large sample study to report of increased ratio of Glu/PCr+Cr in the mPFC among MAD subjects, although preclinical studies have found such findings (51–53). Glu is the major excitatory neurotransmitter, and most of Glu is present intracellularly. Extracellular Glu released from nerve terminals is taken up by Glu receptors and Glu transporters present presynaptically, postsynaptically and extrasynaptically in glial cells, preventing Glu excitotoxicity (54). Glu in the glial cells is converted to glutamine (Gln) through an ATP-dependent process in mitochondria. Gln is subsequently released from the glial cells and taken up by Gln transporters in neurons. Gln in neurons is converted back to Glu. This is the “glutamate–glutamine cycle,” which represents 40–50% of the total flux from the TCA cycle (54). Therefore, dysfunction of Glu receptors or Glu transporters may be a possible explanation for elevated Glu concentration in the mPFC. The elevated Glu concentration suggests that the Gln converted from Glu is decreased and decreased activities of glutamateric neurons. Meanwhile the increased extracellular Glu concentration is neurotoxic.

In the research conducted by Sailasuta et al., around 36% of normal glial tricarboxylic acid (TCA) cycle rate was significant reduced in frontal brain of abstinent MA abusers, which may impact the glutamate-glutamine cyle and thus result in accumulation of Glu (55). Therefore, the other possible explanation of the increased Glu concentration in our study is the consequence of dysfunction of glial cells.

Furthermore, the increased extracellular Glu concentration is considered as an important factor of relapse. Glutamateric signal system plays an important role in drug-seeking behavior. It has been reported that the activation of Glu transporter (56), or gene expression of the transporter in the nucleus accumbens (57) plays an inhibitory role in MA conditioned place preference (CPP), while mPFC is acritical region for reactivation of the MA-CPP memory (58). Glu released from the mPFC stimulates dopamine release in the ventral tegmental area and the nucleus accumbens (59), while dopaminergic system is considered as primary mechanism initiating drug reinstatement (60). In a preclinical study, the increased expression of the Glu transporter attenuated the MA-CPP, which reduced drug-seeking behavior (61).

### Limitations

This study has several limitations. First, most of subjects in this study were men, making it impossible to explore the influence of gender confidently. Second, the characteristics of MAD subjects in this study, including the range of duration of MA use and abstinence, and the drug used in the abstinence would be possible confounding factors. Third, the study is cross-sectional, it is unclear whether alterations in these metabolites would reverse completely during continued abstinence or would persist. Furthermore, MAD subjects had a higher CPD than the controls, and this difference was significant. We noted this difference, controlled this covariate in statistical analysis process and discussed their possible impacts on our MRS results. We concluded that this difference in CPD between the MAD subjects and healthy controls is unlikely to be the reason for the statistically significant alterations of metabolites of the mPFC. Our study is a relatively large sample and we controlled the cigarette smoking when comparing the metabolites. Finally, there were significant differences between MAD subjects and controls in the S/N ratios and CRLB values. But the S/N ratios and CRLB values in both groups characterize relatively good quality of our data. Future studies should include more women MAD subjects, measure alterations longitudinally when MAD subjects using MA (if possible), at the beginning of abstinence, and after longer duration (6–24 months) of abstinence, and match subjects' cigarette smoking.

## Conclusion

Our findings suggest that the alterations in ratios of Glu/PCr+Cr of the mPFC may underlie the pathophysiology of neurological injury in MA abuse. MA cause the Glu concentration elevation, which has neurotoxicity and may lead to NAA concentration decreased and mI increased. This study implicates that Glu plays an important role in MA dependent disorder, reducing Glu concentration or increasing the activity of Glu receptors in the mPFC may be of great clinical significance in the treatment.

## Author contributions

YL, JT, JLi, and TL contributed conception and design of the study. QW, CQ, and AX organized the database. QW performed the statistical analysis and wrote the first draft of the manuscript. JLo, YL, XW, YT, and JT revised the manuscript. TL, BY and WH advised on the statistical analysis, interpretation of findings, and reviewed drafts of the manuscript.

### Conflict of interest statement

The authors declare that the research was conducted in the absence of any commercial or financial relationships that could be construed as a potential conflict of interest.
